# The Healthier the Tastier? USA–India Comparison Studies on Consumer Perception of a Nutritious Agricultural Product at Different Food Processing Levels

**DOI:** 10.3389/fpubh.2016.00006

**Published:** 2016-01-28

**Authors:** Laurette Dubé, Hajar Fatemi, Ji Lu, Cristian Hertzer

**Affiliations:** ^1^Desautels Faculty of Management, McGill University, Montreal, QC, Canada; ^2^Faculty of Agriculture, Dalhousie University, Halifax, NS, Canada

**Keywords:** food, processing, branding, health, taste, natural, culture

## Abstract

Present research compares food beliefs associated with a naturally nutritious agricultural product (namely pulses) in Western and Eastern cultures (namely the US and India). Specifically, this paper focuses on the perception of healthiness and tastefulness of the food and their relationship. Two studies tested the effect of processing level, cultural differences, and branding strategies. In contrast to the well-established inverse relationship between healthiness and tastefulness beliefs observed in the West with industrial food products, the results of both studies revealed a positive association between health and taste for pulses in both West and East. Study 1 shows that this positive association is stronger with lower processing, suggesting the role of naturalness as bridge between health and taste. Focusing on cultural differences, results show that while both West and East hold positive association of health and taste for pulses, this association is stronger for East. However, the role of processing level is significantly stronger in West. Study 2 looks at branding strategies for pulse products with different processing levels in West and East. Results confirm the findings of study 1 on positive association of taste and healthiness and cross-cultural differences. Moreover, study 2 shows that cultural difference between West and East changes the effect of branding strategies on food-related belief and attitude toward food. For American consumers, a future-oriented branding is associated with an enhanced positive healthiness–taste association, whereas a brand image emphasizing tradition leads to increased perception of the taste of product but not necessarily on the healthiness. Current paper has theoretical and practical implications in public policy, health, and marketing.

## Introduction

Taste and healthiness are among the most important attributes of food that impact consumers’ choice and decision making (1–3). Abundant consumer studies conducted in industrialized markets in Western cultures have shown that in consumers’ mind, a food perceived as healthy is often perceived as having poor taste, and conversely, a tasty food is typically perceived as being unhealthy (4–9). Such bipolar beliefs have been observed at the level of food attitudes, choice (10), and eating behavior (11), reflecting a broader tendency of Western cultures to oppose pleasure, hedonism, and other facets of human experience, with health, convenience, and other utilitarian functions of consumer products in general (12, 13). Such bipolar beliefs have adverse effects on multi-facets of consumers’ food consumption, as research has shown that consumers often view healthiness and pleasure as tradeoffs while making food choices (7), and they perceive healthy food less fulfilling (14). It also has been demonstrated that food branded as healthy are ironically associated with higher caloric intake because consumers tend to underestimate the calorie content of the food labeled as “healthy” (15).

As cultural heritages importantly impact food beliefs (16), the bipolar beliefs of taste and health in Western culture do not necessarily exist in Eastern culture. Studies generally suggest that Eastern cultures, such as Chinese and Indian, believe that taste is intrinsically embedded in healthiness, therefore, associate healthiness and tastefulness in a positive way. Cervellon and Dubé (4) found that while an inverse correlation of −0.54 between health/pleasure (hedonic/utilitarian) attributes was consistently found in the Western cultures, such relations was not significant in Chinese respondents. In Chinese culture, such as in India, Japan, and most other Eastern cultures (17), taste and nutrition-related attributes are equally important components of a holistic approach to well-being, thereby invalidating the bipolarity as structuring people’s mindset in their relationship to food (13, 17–19). Specifically in Indian culture and in traditional Hindu system of medicine known as Ayurveda, foods that are pleasant and fresh in taste are the same ones that promote health, physical strength, and mental abilities, whereas those bitter or unpleasant in taste fall in the category of junk or preserved foods (17).

Beyond cultural heritage, difference in the intrinsic quality of food prevailing in Western and Eastern markets is another putative explanations for the empirical results mentioned above. The bipolar beliefs of “unhealthiness = tasty” observed in Western culture are partly rooted in “fun = unwholesome” intuition (7), and also importantly rooted in consumers’ beliefs regarding the manufacturing and marketing of modern processed food. While Western foods have capitalized on industrial ingredients and processing methods to add value to agricultural product, they typically raised the content of fat and sugar, components associated with “superior” taste perception (20). The industrial processing also add value to agricultural commodities by improving the qualities that well adapted to modern lifestyle, including but not limited to convenience and financial accessibility (21). Major investment has also been typically deployed in advertising, in-store promotion, branding, and other marketing practices to reinforce consumer appeal particularly on the hedonic experience (22–26).

Cross-country studies showed advertising and branding of modern industrial Western food are linked to public health problems, obesity, diabetes, and cardiovascular diseases (27–31). With the spread of Western food and food processing technologies to less developed Eastern countries, Western food beliefs and the maladaptive food choice reported above are now diffusing, at an accelerating rate, to countries around the world (32). Question arises as to whether such Westernization path that has prevailed since the beginning of the industrial revolution three centuries ago is the only course that can be taken in moving from tradition to modernity. In earlier work, we have argued that this does not have to be the case (33, 34) and empirically demonstrated that taste and health do not necessarily have to be bipolar opposite, even in Western culture. Fatemi and Dubé (35) found that healthy foods can be perceived as tasty particularly when they are perceived as natural and, even more so, when they consist of naturally nutritious agricultural commodities or minimally processed. Regardless of the nutritional composition of the end product, industrial processing is generally perceived as the opposite of naturalness (36, 37). As humans have “an innate desire for the experience of their ancestral environment” (36), a “natural preference” is observed across different cultures (36) and product domains (38). In food domain, this preference is closely tied to a bridging effect of naturalness on the health–taste bipolar beliefs, that is, natural food is positively evaluated not only on its healthfulness but also on the attributes related to pleasure and other esthetic experiences (36, 39).

Eastern developing countries are still largely operating traditional social system; significant efforts have been made to modernize the agri-food system with a strong focus on addressing undernutrition issues. At the same time, maladaptive food choices that similar to the Western societies are emerging as a public health problem. In order to shed light on the path to modern food system in developing countries, it is important to study the consumption patterns of food, whether it is natural food or processed industrial food (40). Meanwhile, the consideration of traditional food system is also relevant in Western culture, where a trend of returning to traditional/natural food is reflected in the increasing popularity of local food, organic agriculture, and natural products (36–38). However, the scale of these transitions is still inadequate to solve maladaptive diet and health problems in Western developed countries.

In this paper, we suggest that in both Western and Eastern cultures and markets, taste and health do not have to be polar opposites when food with different levels of processing are anchored into naturally nutritious agricultural commodities. We report two studies in which we have taken pulses (e.g., chickpeas and lentils) as the proof of concept for naturally nutritious agricultural commodities and proceeded to a cross-country comparison between the US and India. Global health and food agencies, as well as US and Canadian health agencies, have recommended pulses as a healthy and nutrient choice of food and as part of a healthy diet (41). Beyond their protein and fiber content, pulses are known to have many health benefits, including blood pressure control, reduced risk of obesity, diabetes, and cardiovascular diseases (42). In order to improve global market for pulses, the promotion efforts have to consider the interplay of regional and national level cultural differences, along with exceptional characteristics of pulse products and various branding strategies to advertise these products. This study is a step forward to achieve this goal.

## Methods and Results

### Overview of the Studies

Both studies were conducted online through Amazon Mechanical Turk (AMT), which is an online survey platform widely used for consumer research and other social studies (43). Required ethics approval was obtained from university research ethics board. Indian and US participants were recruited through AMT and received $2 for completing this study. The cultural background of the participants was determined by their self-report on the place of living and IP address from where they answered the questionnaire. Self-reported demographic information of both studies’ samples of Indian and US participants are shown in Table 1. In both studies, compared to US participants, more Indian participants were male, younger, lived in large cities, and lived with larger number of family members.

**Table 1:** **Demographic descriptions of Indian and US participants in Studies1 and 2 and Chi-square tests comparing US with Indian participants**.

	Study 1		Study 2	
	India (*n* = 204)	US (*n* = 204)	India (*n* = 305)	US (*n* = 304)
Gender	Female = 41%	Female = 49%	Female = 36%	Female = 47%
	Chi-square(1,408) = 2.96; *p* = 0.1		Chi-square(1,609) = 7.54; *p* = 0.01	

Age				
20 or less	0%	4%	2%	4%
21 to 30	56%	47.5%	55%	33%
31 to 40	28%	29%	30%	35%
41 to 50	10%	12%	10%	14%
51 or more	6%	7.5%	3%	14%
	Chi-square(4,408) = 10.22; *p* = 0.04		Chi-square(4,609) = 35.95; *p* < 0.001	

Place of living				
Large city	48%	27.5%	46%	23%
Middle size town	31%	41%	29%	43%
Small town	19%	23%	18%	27%
Rural village	2%	8.5%	7%	7%
	Chi-square(3,408) = 21.15; *p* < 0.001		Chi-square(3,609) = 34.90; *p* < 0.001	

Primary person grocery shopper	Yes = 78%	Yes = 86%	Yes = 74%	Yes = 85%
	Chi-square(1,408) = 4.32; *p* = 0.04		Chi-square(1,609) = 11.57; *p* = 0.001	

Living with family	Yes = 96%	Yes = 65%	Yes = 95%	Yes = 68%
	Chi-square(1,408) = 60.93; *p* < 0.001		Chi-square(1,609) = 74.93; *p* < 0.001	

Number of people in family				
3 or less	38%	70%	30%	70%
4 to 6	59%	30%	60%	28%
More than 6	3%	0%	10%	2%
	Chi-square(3,408) = 122.3; *p* < 0.001		Chi-square(3,609) = 176.21; *p* < 0.001	

In both studies, all the participants read the description of six pulse-based products. The six pulse products were at three different processing levels. Participants were asked to report their perceptions of each product in terms of its taste and healthiness. In study 1, we explored, in both Indian and US cultures, how the healthiness and taste beliefs were differently aligned in healthy agriculture product that form traditional/natural food or used as ingredients in industrial processed product. In Study 2, we looked at the ways modern branding strategies promoting innovation/naturalness were used in both cultural contexts to reduce or prevent the development of bipolar healthy = not tasty belief systems. Specifically, comparing Western and Eastern cultures, this paper explored three potential branding strategies along the past-now-future continuum, with emphasizes on innovative technology, current situation, and tradition food production method, respectively. For each study, we first briefly laid out the research rational and expectation followed by study description and results. The general findings and implementations are discussed at the end.

### Study 1

#### Rational

We propose that, within an inherently healthy agricultural product category, such as pulses, the healthiness–taste association may be different from the unhealthy = taste association. Studies revealing such bipolar beliefs in Western context typically compare different categories of agricultural products or products with different ingredients without controlling for the processing level. For example, it has been found particularly for US consumers that “tasty” words are associated with unhealthy processed foods, such as pizza and French fries, as compared to fresh fruits and vegetables [2006; (44)]. Some other studies found unhealthy = tasty association by comparing products at the same processing level with healthy versus unhealthy ingredients [e.g., Raghunathan et al. (7)], and such manipulation of healthiness intrinsically interferes taste perception. Considering the potential shortcomings of previous literature, current study focuses on a single food, i.e., pulses that are biologically neutral in taste (45). Keeping this naturally nutritious agriculture product constant, current study explores consumers’ healthiness/taste perceptions in various processing levels, from natural form (homemade dishes) to industrial processed products.

#### Experiment Design and Procedure

Four hundred and eight participants from India and the US (*n* = 204 for each country) were hired through AMT. The stimuli used in both studies were descriptions of six hypothetical pulse products varying in level of processing. The six products were different in their level of processing. There are three processing levels, from unprocessed to minimal processed to processed, with two products instances in each level (see Table 2 for the stimuli descriptions). At the unprocessed level, the two products were homemade lentil and veggie salad and Homemade Chickpea and Oat Cookies. The descriptions of products emphasized that the “Whole Pulses are used for home cooking.” At Minimal processed level, the two products were prepackaged products made from whole pulse ingredients (or flour, flakes), for example, bottled chickpea and berry smoothie. At processed level, the two pre-package products are made from pulse-based processed ingredients, including pulse protein and fiber, such as prepackaged lentil and fruit granola bar.

**Table 2:** **Pulse product stimuli used in Studies 1 and 2**.

**Level 1: Unprocessed: using pulse produce for home cooking**
(s1) Homemade Lentil and Veggie Salad: whole pulses sometimes are used for home cooking. This is a mix of lentils (cooked), olives, onion, tomatoes, green peppers, cucumber, feta cheese, and parsley, blended with whisk oil and lemon juice
(s2) Homemade Chickpea and Oat Cookies: whole pulses sometimes are used for home cooking. These cookies are baked with Chickpea, whole wheat flour, rolled oats, egg, and baking soda, mixed with canola oil and brown sugar
**Level 2: Minimally processed: using whole pulse (or flour, flakes) as ingredients**
(s3) Bottled Chickpea and Berry Smoothie: whole pulses sometimes are used in processed food products. This bottled juice smoothie product is processed by blending whole chicken peas with fruits, including sweet cherries, strawberries, plum, and apple
(s4) Prepackaged Lentil and Rice Chips: whole pulses sometimes are used in processed food products. This prepackaged bean snack is processed by baking the blended mix of whole Lentils, whole grain long brown rice, pure sunflower oil, and sea salt
**Level 3: Processed: using processed pulse products (fiber or protein) as ingredients**
(s5) Prepackaged Lentil and Fruit Granola Bar: processed pulse ingredients (fiber or protein) sometimes are used in food formulation. This prepackaged nutrition bar contains Lentil protein and fiber, green lentil flakes, pea flour, black bean flakes, red bean flakes, and red lentils
(s6) Prepackaged Chickpea Fiber Breakfast Cereal: processed pulse ingredients (fiber or protein) sometimes are used in food formulation. Fortified by Chickpea fiber, this prepackaged breakfast cereal contains whole grain corn, sugar, and wheat starch

The six products were presented to participants in random orders. For each product, participants answered questions (anchored 1–10, from “not at all” to “very much”) regarding the healthiness perception (“This is a healthy food item.”) and taste perception (“This is a tasty food item.”). Participants also indicated their perception of naturalness of each product (three items, Cronbach’s Alpha = 0.75).

#### Statistical Analysis

To investigate the relationship among healthiness, taste perception, and processing level of the products as well as the moderating effect of culture, mixed models of repeated measure were applied. For model estimation and hypotheses testing, we adopted bootstrapping method. The adopted bootstrapping process draws samples with replacement from the original dataset and computes the estimators for each of the samples. Such procedure was repeated 1000 times. From these 1000 bootstrap estimates, an empirical distribution of the estimators can be inferred. The hypotheses then can be tested based on this empirical distribution. The bootstrapping procedure is distribution independent, through which estimations can be made without the assumption of a normal distribution of dependent variables (46).

In the first model, healthiness perception for each product (408 participants × 6 products = 2448 observations) was predicted by processing level (three-level within-subject factor: unprocessed, minimal processed, and processed), culture (two-level between-subject factor: the US vs. India), and their interaction, controlled by the familiarity of pulse reported by each participant (positive relationship, *p* < 0.001) and the interaction between familiarity and culture (not significant). Participants’ gender and age were also included in the model as control variables (not significant ps > 0.40).

Analysis on taste perception is to test the relationship between the perceptions of healthiness and taste as well as the moderating effect of culture and processing level. A mixed model of repeated measure similar to the analysis on healthiness perception was conducted to explain the variation of taste perception (factors: culture, processing level, and their interaction). The model further included healthiness perception as a predictor of taste perception to explore the healthiness–taste relationship. The interaction of culture by healthiness perception was included to examine the moderating effect of culture on healthiness–taste correlation. A three-way interaction of culture, healthiness perception, and processing level was further included in the model. Final model controlled for familiarity to pulse (significant, positive) and its interaction with culture. The model also included participants’ gender and age as control variables, which were not significant (ps > 0.19).

#### Results

##### Healthiness Perception

**Figure 1:**
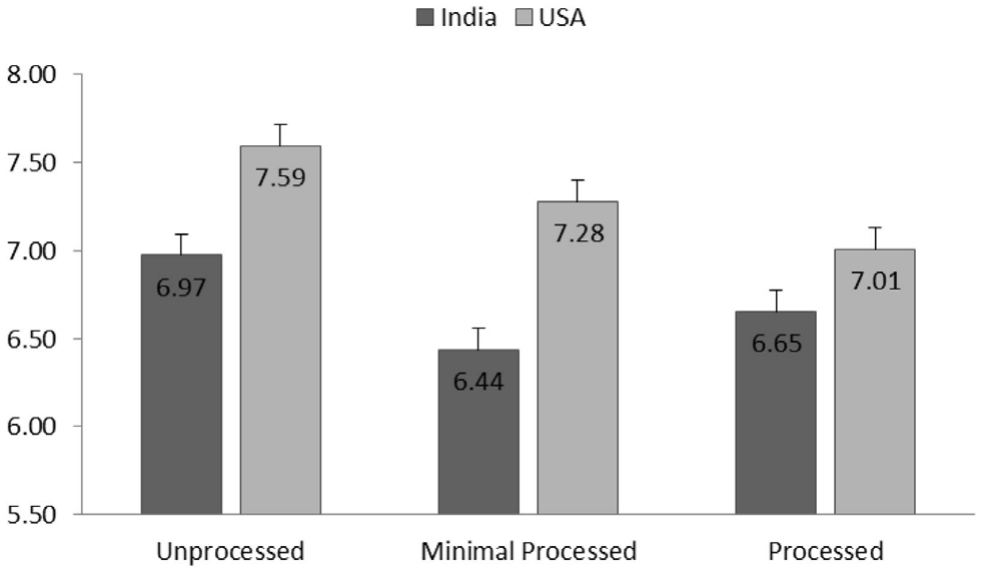
**Group means (SE, as represented by error bars)of healthiness perception for each processing level and separated for American and Indian participants**.

The effect of processing level was significant on healthiness perception [*F*(2,2032) = 26.25, *p* < 0.001], indicating lower healthiness perception for products with higher processing level. As seen in Figure 1, the group means of healthiness perception for each culture demonstrate a general decreasing trend as the product processing level increases. The effect of culture on healthiness perception was significant [*F*(1,402) = 17.09, *p* < 0.001]. Indian participants (*M* = 7.39, SD = 1.81) reported higher healthiness perception for pulse products than American participants (*M* = 6.41, SD = 2.48).

The interaction effect of processing level and culture was found significant [*F*(2,2032) = 4.86, *p* = 0.008]. *Post hoc* group means comparisons [all the multiple comparisons hereafter were adjusted by Bonferroni (47) procedure] suggested that the effect of processing level is stronger for American participants compared to Indian participants. Specifically, American participants reported higher healthiness perception for unprocessed products than processed products (*p* < 0.001). Minimally processed products were also perceived significantly healthier than products in two higher levels of processing (*p* < 0.001 and *p* = 0.03). For Indian participants, the unprocessed food was perceived to be healthier than more processed food (*p* < 0.001) and minimal processed products (*p* = 0.02). However, minimal processed products were not significantly healthier than products in higher level of processing (*p* > 0.5).

##### Healthiness perception

**Figure 2:**
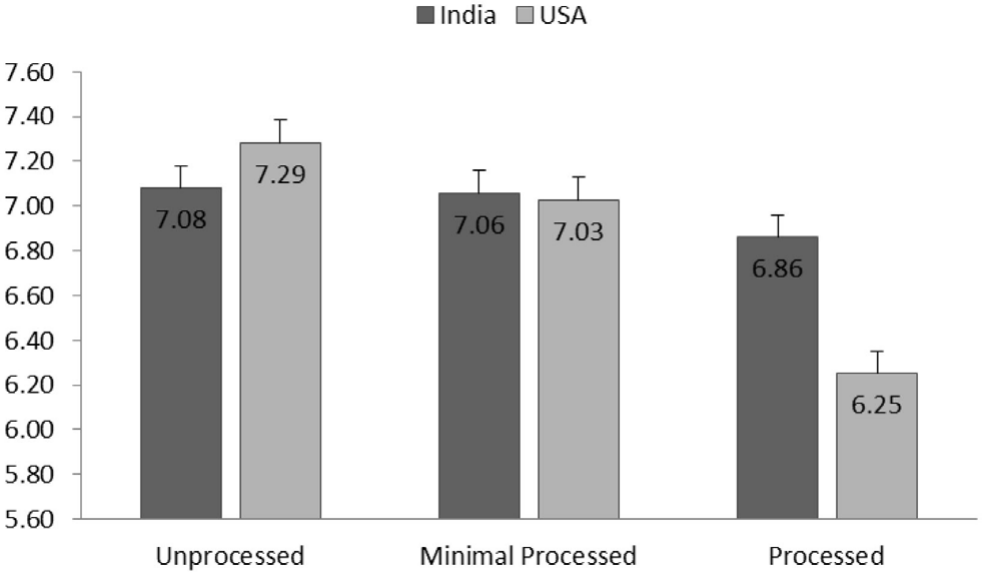
**Group means (SE) of taste perception for each processing level and separated for American and Indian participants**.

Results showed that the effect of culture on taste perception was significant [*F*(1,1936.82) = 5.55, *p* = 0.02]. Average taste perception for all pulse products reported by Indian participants (*M* = 7.39, SD = 1.81) was higher than that taste perception scores reported by American participants (*M* = 6.41, SD = 2.48). The main effect of processing level was significant [*F*(2,2102.59) = 7.64, *p* < 0.001]. Marginal means showed a general decreasing trend for taste perception when the processing level increased (See Figure 2). A significant interaction effect of culture by processing level [*F*(2,2105.25) = 5.20, *p* < 0.01] revealed that the processing level influenced American participants more strongly than Indian participants. Particularly for American participants, difference of taste perceptions across all three processing levels were significant (ps < 0.03), whereas for Indian participants, this difference were not significant (ps > 0.09).

**Figure 3:**
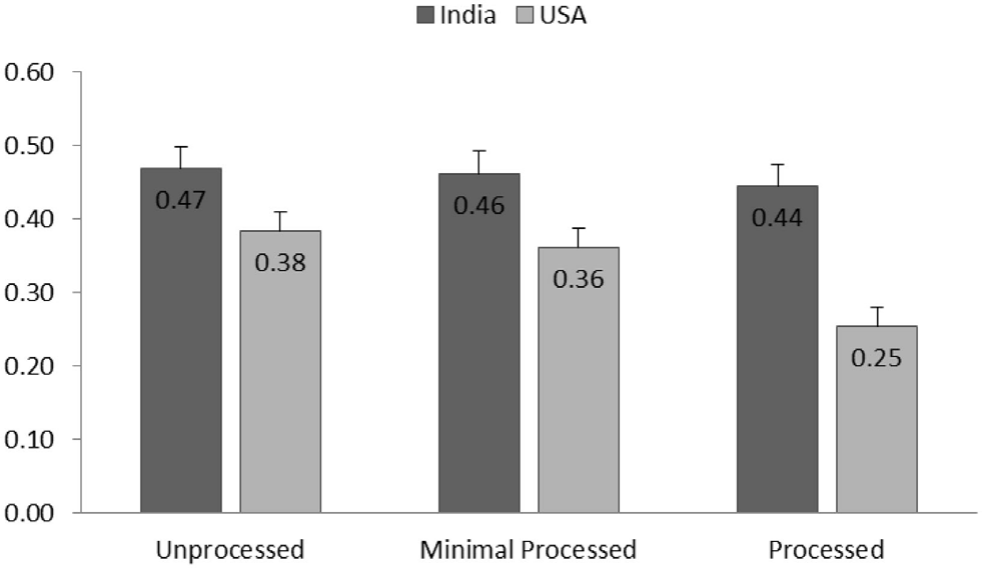
**The estimated coefficients (SE) of healthiness predicting taste (healthiness–taste relationship) separated by cultures and product processing levels**.

Effect of healthiness perception on taste perception was positive and significant [*F*(1,2427.27) = 373.73, *p* < 0.001]. Significant interaction between culture and healthiness [*F*(1,2427.18) = 8.17, *p* = 0.004] indicated that the positive relationship between taste and healthiness perception was stronger for Indian participants (estimated coefficient: *b* = 0.53, *p* < 0.001) than for US participants (*b* = 0.35, *p* < 0.001). The three-way interaction of culture, healthiness, and processing level was found significant [*F*(2,2113.57) = 5.35, *p* < 0.01]. The direction of this effect showed that relationship between healthiness and taste perception was moderated by processing level, and the moderating effect was different for Indian and US culture. Figure 3 shows the estimated coefficients of healthiness predicting taste separated by cultures and product processing levels. As indicated by estimated coefficients and *post hoc* comparisons, the healthiness–taste association decreased as the processing level increased, while such effect was only significant for US participants (all the pairwise comparisons between taste–healthiness association estimated for each processing level, ps < 0.001) but not for Indian participants (comparisons, ps > 0.06).

#### Conclusion

Results of Study 1 reveal that products with a lower level of processing are generally perceived to be healthier and tastier, compared to industrial processed pulse products. For pulse products, healthiness perception and taste perception are positively associated, and such association is stronger in Indian culture than in American culture. Furthermore, only for American culture, a higher level of processing tends to decrease the healthiness–taste correlation.

### Study 2

#### Rational

Branding activities are powerful tools to influence consumers’ perception (48, 49). The healthy food promotion strategy can be fine-tuned for different cultures based on a better understanding of healthiness–taste beliefs and consumers’ reactions to modern and traditional agri-food operations in different cultures. Eastern and Western food system and food beliefs are largely anchored to traditional and modernized operations, respectively. Along this past–future temporal continuum, current study explores three potential branding strategies, emphasizing on innovative technology, current time, and tradition food production method.

A branding strategy emphasizing the “future” often featured as the use of most advanced technologies. To Western cultures, as the bridging effect of naturalness on healthiness and taste perception diminishes in higher degrees of industrial processing, it is interesting to explore the effect of a future-oriented branding strategy on the healthiness and taste perceptions of traditionally consumed healthy food. In Eastern developing countries, innovative technologies are yet new and something to be achieved and valued. As a result, a future-oriented branding strategy may be preferred in developing countries, as reflected in favorable perceptions on taste and healthiness. In light of the fact that 2016 will be the international year of pulses, this investigation on branding strategies are particularly timely, as the 2016 year of pulse takes “future of food” as the slogan for its global marketing strategy.

Alternatively, branding strategy can emphasize past/tradition or current time. Tradition concept is found to be congruent with naturalness (50) bringing together the healthiness and taste perception of food. This is particularly the case in developed Western countries. As compared to Eastern countries, Western society has already experienced technological advances and is more inclined toward getting back into tradition and nature. This approach will make the traditional branding strategy likely to be successful in increasing both taste and health perceptions of food products in West. Current time branding strategy underlines present-day situation. In developed Western countries, it highlights applying present modern techniques and technologies, mainly insisting on industrial processed food. In contrary, current time branding strategy in developing Eastern countries highlights traditional food produced with presently dominant traditional non-industrial technologies. As a result, we expect current time branding strategy to magnify current state of food beliefs; in West to underline bipolar beliefs of health and taste and in East to underline traditional balanced view on healthiness and taste.

#### Experiment Design and Procedure

Study 2 replicated the procedure of Study 1. Participants from the US and India read descriptions of the same six pulse products as in study 1 and answered same set of questions regarding their healthiness perception and taste perception for each product. Six hundred and nine participants from India (*n* = 305) and the US (*n* = 304) were randomly assigned to three experimental conditions, varying in branding strategy: future (US: *n* = 100; India: *n* = 100), current (US: *n* = 104; India: *n* = 103), and past (US: *n* = 100; India: *n* = 102).

Before reading the descriptions of six pulse products, participants read description of a food producing company that produced pulse products along with its logo and its slogan [adapted from Pulse Canada (http://www.pulsecanada.com/)] (see Table 3). In the future condition, the brand was described to have future-orientation on innovative technologies for creating a sustainable environment. In current condition, the brand was pictured as emphasizing on living at the moment, whereas in past condition, the brand was described to value tradition and the past generations.

**Table 3:** **Branding strategies manipulation in Study 2**.

Brand Logo and Slogan	Branding Description
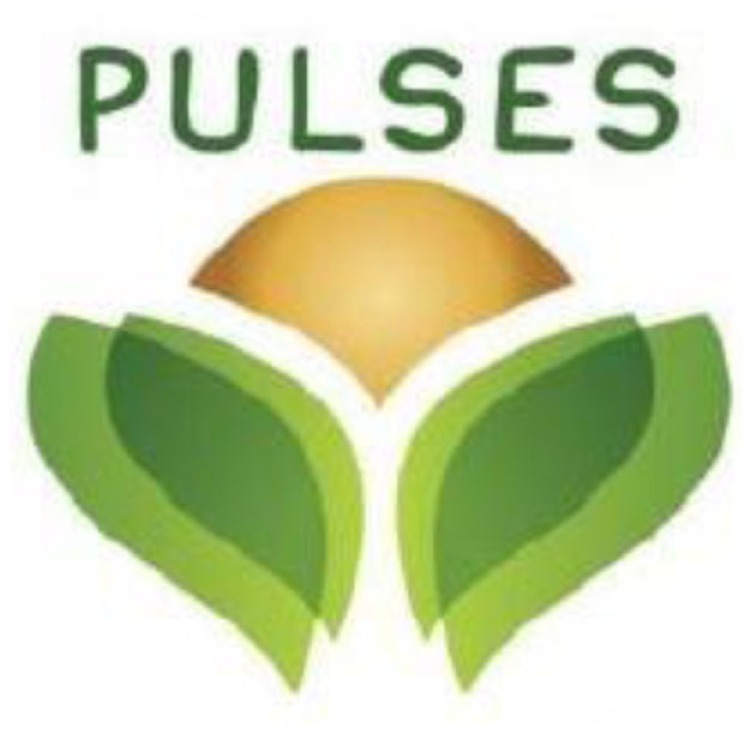 Future of Food	*Future Condition:* in the following, you will see series of pulse foods that are associated with brand A. Brand A (see its logo below) is well-known by its future-orientation. This brand is focusing on innovative sustainable solutions to save the future generations
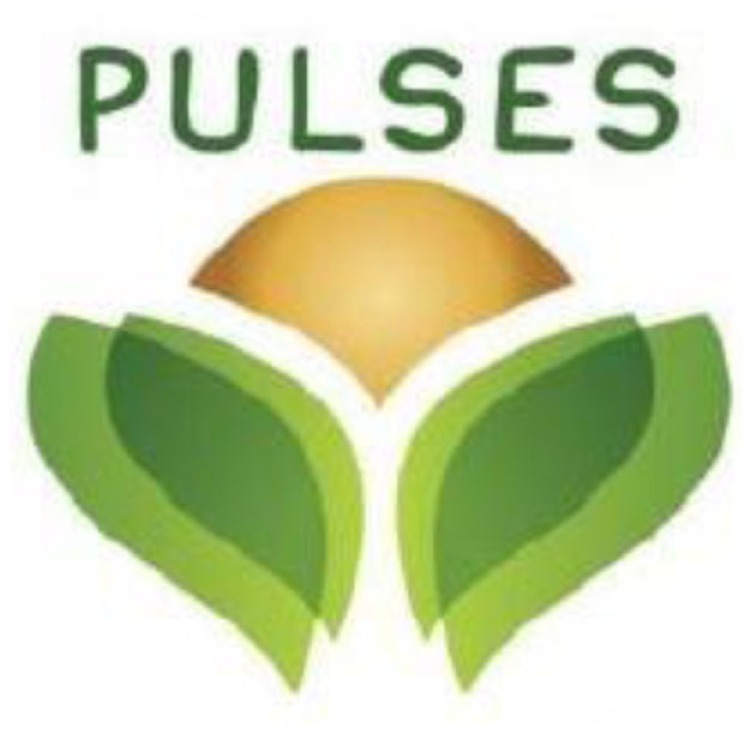 Food of the Day	*Current Condition:* in the following, you will see series of pulse foods that are associated with brand A. Brand A (see its logo below) is well-known by its present orientation. This brand focuses on capturing the present time and living in the moment
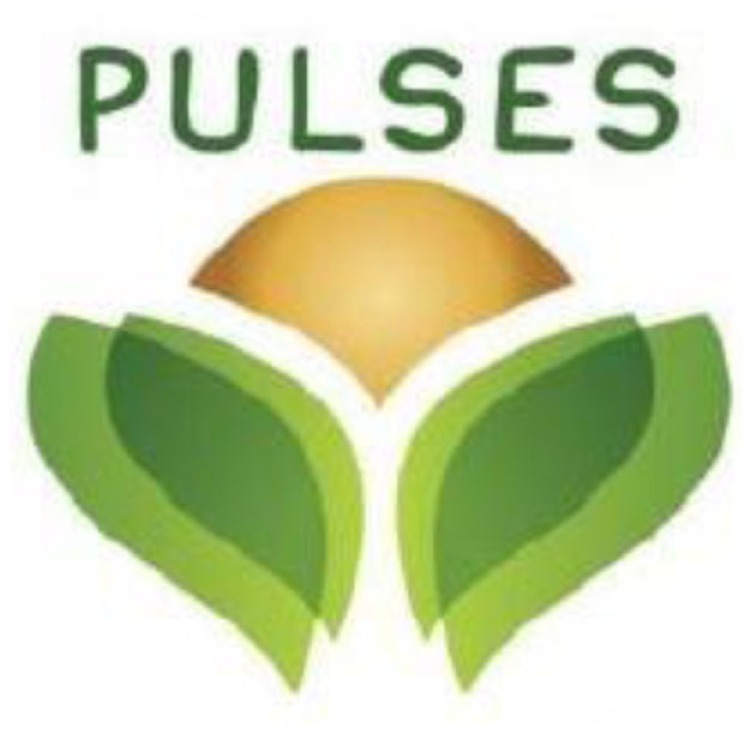 Food of Tradition	*Past Condition:* in the following, you will see series of pulse foods that are associated with brand A. Brand A (see its logo below) is well-known by its past orientation. This brand focuses on traditional foods and links people with previous generation’s foods

#### Statistical Analysis

Similar to Study 1, healthiness perception was analyzed in a mixed model of repeated measure (estimated by bootstrapping procedure). The model included branding conditions (three-level between-subject factor: future, current, and past), culture, processing level, and their interactions as predictors, controlled by personal familiarity with pulses (*p* < 0.001) and its interaction with culture. The model was also controlled with participants’ gender and age (not significant ps > 0.10).

Taste perception was analyzed by a mixed model of repeated measure. It was predicted by culture, branding conditions, healthiness perception, and their interactions, controlled by familiarity with pulses (*p* < 0.001) and its interaction with culture, as well as gender and age (ps > 0.19).

#### Results

##### Healthiness Perception

**Figure 4:**
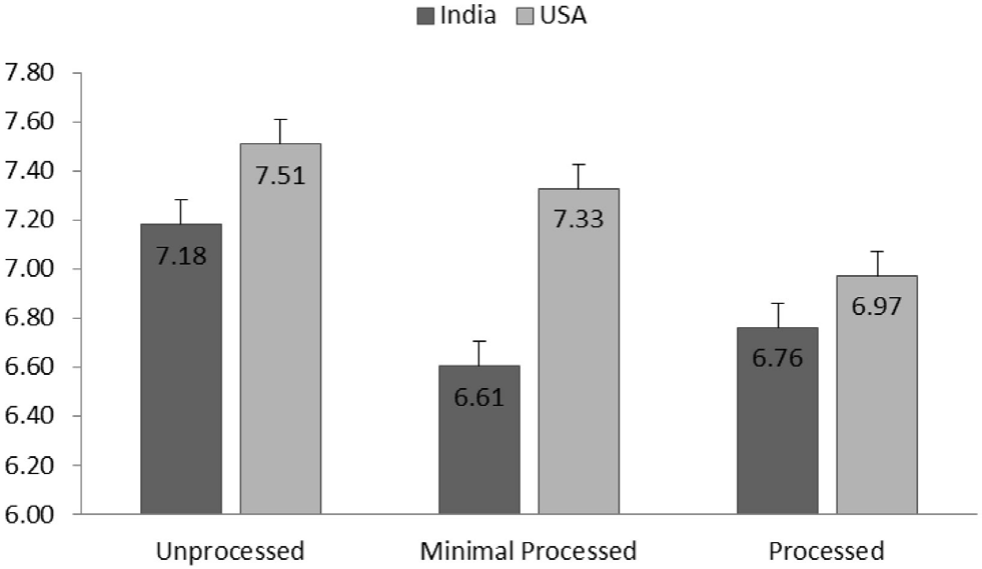
**Group means (SE) of healthiness perception for each processing level and separated for American and Indian participants**.

The effect of culture on healthiness was significant [*F*(1,599) = 5.61, *p* = 0.02]. American participants reported lower healthiness perception toward pulse products (*M* = 7.11, SD = 1.99) than Indian participants (*M* = 7.38, SD = 1.93). Effect of processing level on healthiness perception was significant [*F*(2,3033) = 37.02, *p* < 0.001]. Healthiness perception was lower for products with higher processing levels (See Figure 4). A significant interaction between culture and processing level [*F*(2,3033) = 10.50, *p* < 0.001] was found, showing differential effect of processing level on healthiness perception for American and Indian participants. Specifically, for American participants, the processed pulse products were perceived to be significantly less healthy (ps < 0.001) than unprocessed and minimal processed products, while the difference between latter two were marginally significant (*p* = 0.08). Indian participants perceived unprocessed product to be significantly healthier than products with higher processing levels (*p* < 0.001), and the difference between minimal processed and processed products was not significant (*p* = 0.2).

Non-significant main effect of branding strategy (*p* = 0.59) and all the interactions involving it (ps > 0.5) revealed that the type of branding strategy did not influence healthiness perception of pulse products.

##### Taste Perception

**Figure 5:**
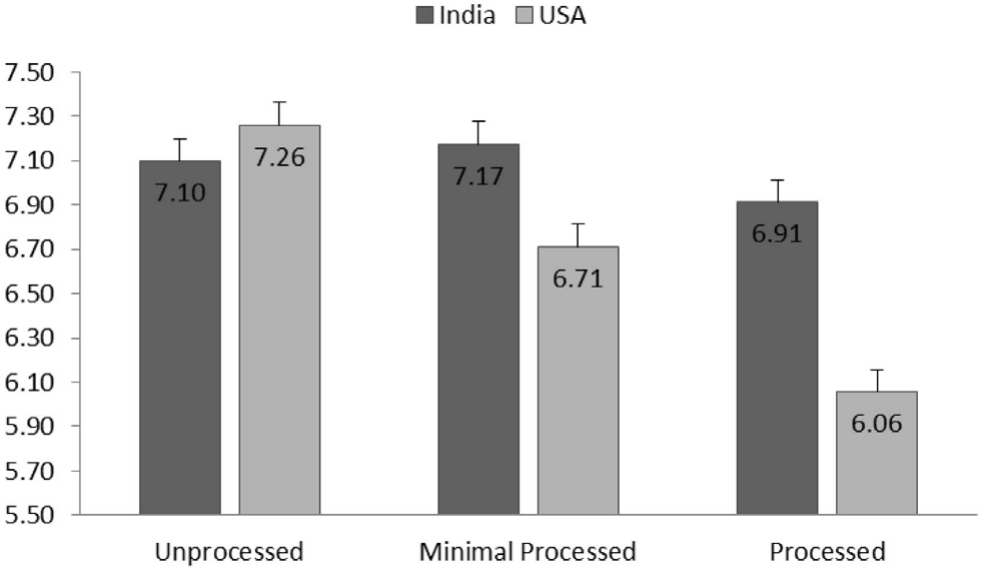
**Group means (SE) of taste perception for each processing level and separated for American and Indian participants**.

The effect of culture on taste perception was significant [*F*(1,2702.49) = 29.28, *p* < 0.001]; US participants reported lower taste for pulse products (*M* = 6.19, SD = 2.39) than Indian participants (*M* = 7.41, SD = 1.90). The effect of processing level [*F*(2,3152.81) = 3.24, *p* = 0.04] and the interaction of processing level and culture [*F*(2, 3154. 14) = 5.21, *p* < 0.01] were significant. The estimated marginal means (Figure 5) indicated that taste perception decreased when processing level increased, particularly for American participants. Within American participants, all the pairwise taste perception comparisons between three processing levels were significant (ps < 0.001). Within Indian participants, only the comparison between minimal processed and processed pulse product was significantly different in terms of taste perception (*p* < 0.01).

**Figure 6:**
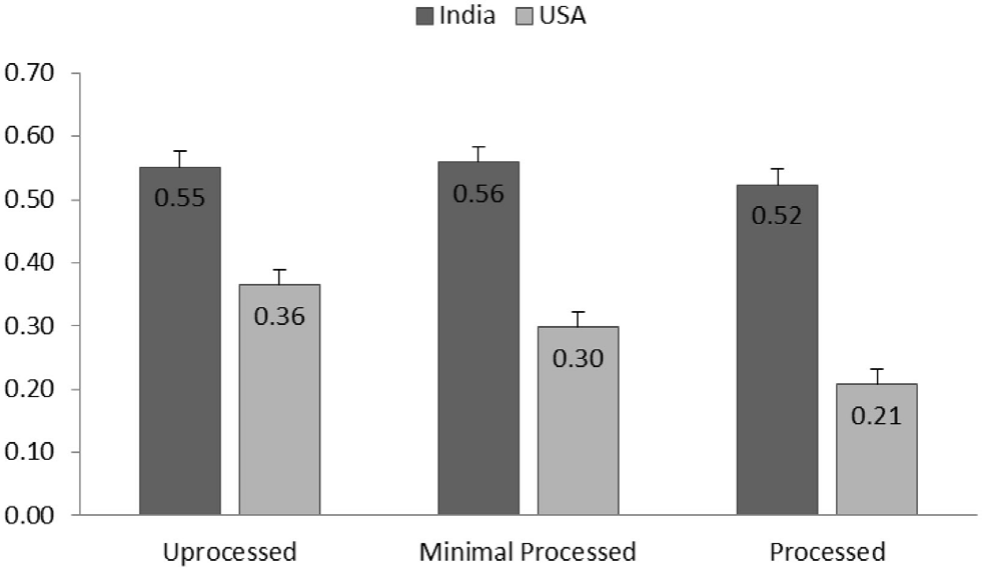
**The estimated coefficients (SE) of healthiness predicting taste (healthiness–taste relationship) separated by cultures and product processing levels**.

Healthiness perception was found to be positively associated with taste perception [*F*(1,3609.50) = 669.91, *p* < 0.001]. The interaction of healthiness and culture [*F*(1, 3609.85) = 61.50, *p* < 0.001] showed that Indian participants’ healthiness–taste correlation was stronger than that of Americans. A three-way interaction between culture, processing level, and healthiness revealed that the healthiness–taste relationship was moderated by both processing level and culture. As showed in Figure 6, in Indian culture, the healthiness–taste association was not significantly different across all processing levels, whereas for American participants, the healthiness–taste relationship was significantly different and weaker for products with higher processing level (all the *post hoc* parameter comparisons were significant; ps < 0.05).

**Figure 7:**
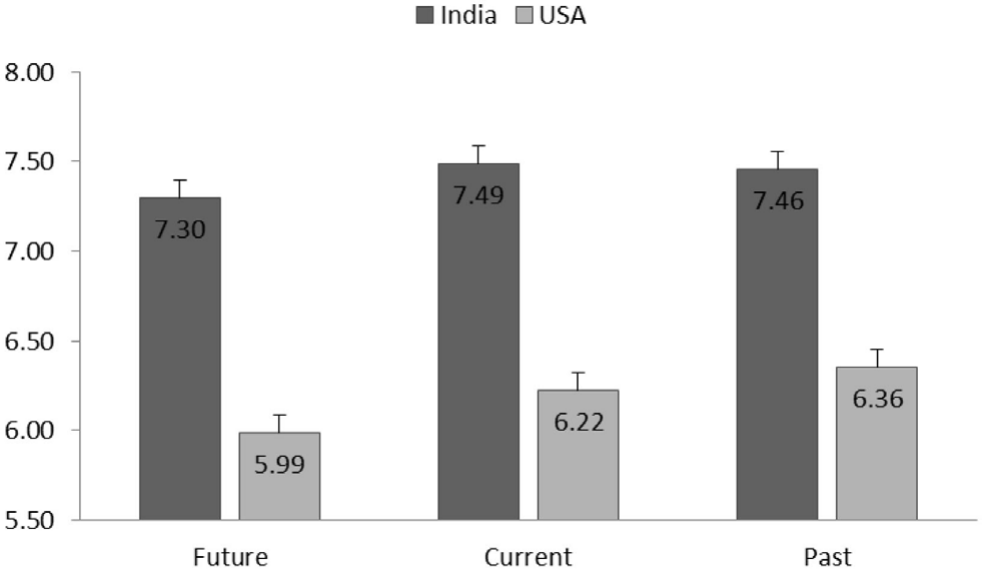
**Estimated taste perception (SE) reported by participants from different cultures and branding conditions (future, current, and past)**.

The effect of branding strategy on taste perception was significant [*F*(2,2993.82) = 4.02, *p* = 0.02]. Furthermore, a two-way interaction between branding strategy and culture [*F*(2,2996.11) = 6.24, *p* = 0.002] demonstrated that the impact of branding strategy was different in the US and India. Effect of branding condition on taste perception was particularly strong for American participants [*F*(2,596.61) = 2.87, *p* = 0.05], whereas the taste perceptions reported by Indian participants in the three branding conditions were not significantly different (*p* = 0.7). Among American participants, those in past condition reported the highest taste perception (see Figure 7), which was significantly higher than those reported in the future condition (*p* < 0.05).

**Figure 8:**
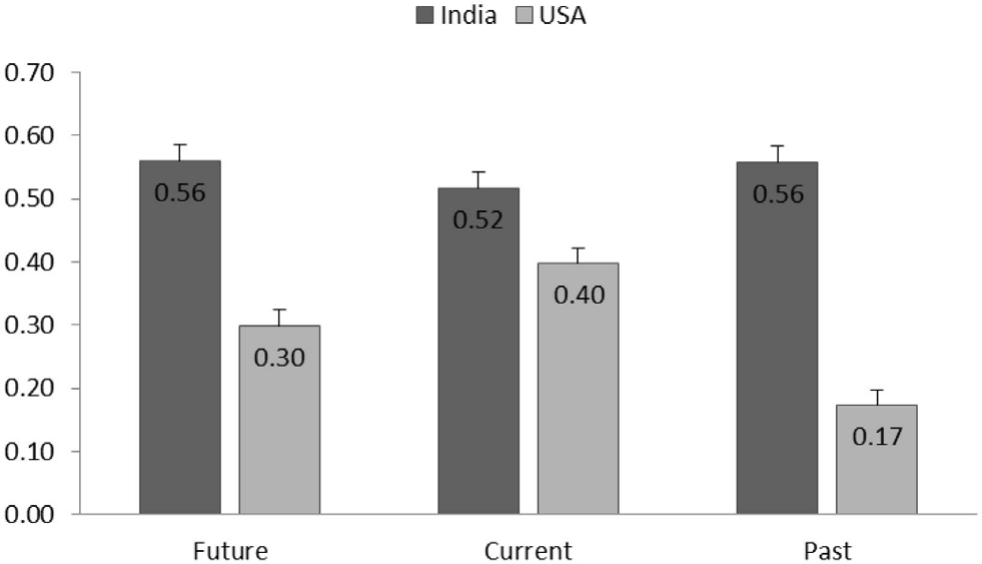
**The estimated coefficients (and SE) of healthiness predicting taste (healthiness–taste relationship) separated by cultures and branding conditions (future, current, and past)**.

The interaction of branding strategy with healthiness perception and culture was found significant [*F*(4,3563.51) = 5.54, *p* < 0.001], indicating that branding strategy moderated the healthiness–taste association and this effect was different for Indian and American participants. Figure 8 demonstrated that Indian participants’ healthiness–taste association was not significantly different in branding strategy conditions (ps > 0.09), whereas the branding strategy significantly moderated American participants’ healthiness–taste association. Specifically among American participants, the healthiness–taste association was the weakest in the past condition and it was significantly lower than the other two conditions (ps < 0.01).

#### Conclusion

Results of Study 2 replicate the findings in Study 1. For pulse products, healthiness is positively associated with taste, while stronger healthiness–taste association was observed in Indian culture and for more natural products. Americans, in contrast to Indians, demonstrated a stronger difference in healthiness–taste association for products in different processing levels. Study 2 highlights that a branding strategy focusing on past, particularly for American participants, is associated with increased taste perception and decreased healthiness–taste association. This result suggests that for Americans, a brand image emphasizing tradition may lead to increased perception of the taste of product but not necessarily on the healthiness.

## Discussion and Future Research

This paper extended the work on food decision making, specifically on the association of taste and healthiness and the impact of culture and branding strategy on this association. Current study is the first one to look at the interaction of taste and health of a single nutritious, healthy food item, i.e., pulses. Unlike prior literature that mainly compared industrial healthy vs. unhealthy food products, current paper looks at one healthy product and studies consumers’ perceptions of health and taste of this product in various processing levels. Our results mainly show the positive association between healthiness and taste for pulses, as healthy traditional food products. This finding is in contrary with unhealthy = tasty association found in prior studies in Western population. We attribute this contradiction to type of stimuli, i.e., industrial unhealthy and healthy stimuli in previous vs. different processing levels of one healthy non-industrial stimulus in current study. The positive relationship between healthiness and taste indicates that, at least for some inherently nutritious products, a healthier alternative does not necessarily always lead to negative perceptions on the sensory attribute. This is another piece of empirical evidence showing that taste and health do not necessarily have to be bipolar opposite, even in Western culture. Nevertheless, our results consistently found stronger positive healthiness–taste association for Eastern vs. Western population. This is in line with the difference between Indian and American food belief systems; Indian culture generally hold a holistic view to the sensory property and nutrition (17), whereas Americans often believe a long-term health benefit is at the cost of short-term hedonic enjoyment (51, 52).

Aligned with findings of prior literature on bridging effect of naturalness on taste and healthiness (36), we found that positive association of healthiness and taste decreases in products with higher level of processing. More extensive use of industrial processing leads to lower evaluation of naturalness of product that results in decreased perception of both sensory and nutritional attributes of food. Although homemade and industrial packaged foods share similar major ingredients, homemade food is more aligned with ideological picture for naturalness; hence, the bipolarity of healthiness and taste is better bridged, leading to better taste and healthiness.

Looking at cross-cultural differences, we found significant effect of processing level in West, with no such effect in East. This finding is in line with the cultural difference between West and East, mainly regarding their different approaches on technological development in food industry. Americans have relied on modernized food technologies and industrialized food products for decades. Recently, however, industrialized food system has been blamed for major contributors to the maladaptive eating habit along with environmental negative impacts. Western consumers are becoming increasingly skeptical to the advances in food and agriculture technologies and more in favor of purchasing natural, organic, and local food. Compared to American, Indian participants were less sensitive to the processing level of pulse products. In a developing country, such as India, industrialized food supply system, as reflected in packaged food, supermarket, and global brand names, is considered as a symbol of modernization and better living standard. As a result of this view, negative evaluation of increased processing level was found in our two studies. These results shed light on the promotion of naturally nutritious agricultural commodities in different cultural contexts. As Western and Eastern consumers tend to have different perception to the industrial processing, the promotional effectiveness would be different depends on either promoting the product as the ingredient used in industrial food or as commodities in its natural/traditional form to be used in home cooking.

Study 2 found branding strategies to moderate consumers’ perceptions of food products. This effect, however, was found only mainly in West and not in East. For American participants, a brand image emphasizing tradition (past condition) or present situation (current condition) was associated with increased taste perception, as opposed to innovation-oriented marketing strategy (future condition). In line with our expectation, American participants valued return of “old time” traditional agri-food production. Steady growth of organic farming, farmer’s market, and storytelling packages is the result of this change in values. By contrast, they negatively valued future looking modern industrialized innovations. Positive perceptions of products in current condition might be linked to dominant American fast-paced daily living culture. In this type of culture, a brand strategy focusing value of now and immediacy gains positive response from customers. We did not find significant effect of branding strategy in India. This might be explained by the fact that India as a developing country is still at transition phase, moving from tradition to innovation. While innovations and industrialization are valued, tradition is still an undeniable dominant part of Indian lives. As a result, positive and negative effects of focusing on future innovations, current situation, and past traditions seem to balance so that we found not significantly different taste perception for products in all three branding strategies.

Contrary to our expectation, we did not find significant effect of brand strategy on healthiness perception. One possible explanation for this finding is that the nutritious nature of pulse sets a general “brand image” for all products based on it, and the processing level effects are relatively strong enough to cause the variation of healthiness perception making branding effect less detectable. Further analysis is required to verify this explanation. Nevertheless, in West, the positive healthiness–taste association was weakest when pulse products were produced by a firm with tradition orientation, as compared to future and current conditions. This result can be attributed to the fact that traditional producing techniques are generally not evaluated to be necessarily healthier than innovative industrial techniques. As a result, a tradition brand image can lead to a higher perception on sensory attributes but not on healthfulness attributes.

Findings of current study are limited in several aspects, suggesting avenues of research for future studies. First, both studies of current paper are online questionnaires, asking for perception of taste and healthiness of pulse products. Results will be more realistic if a real tasting experience in lab environment or in a field study can examine the findings of this study. Also, the study is based on online experiments. Specifically in India, internet access is not conveniently available for all population; thus, this study might be only representing a portion of the population of India particularly biased to younger generation living in large cities. Second, current paper does not look into real consumption and purchasing behavior. Analyzing actual sales data of various pulse products in different processing levels can provide useful insights regarding the interaction effect of product perception, cultural differences, and branding strategies on actual purchase behavior of consumers. Third, as consumers have become increasingly conscious about source of their food, agriculture systems are changing to become one of the main influencing factors in food product perception. Popularity of food products from local farms and fair trade sources are examples of this effect. Current study has not looked into the effect of agriculture systems in product perception. In terms of pulse products, as they are mainly consumed in low processed form, effect of agriculture systems would be more pronounced. Future research may look at this effect, comparing pulse products produced by local farms versus multi-national firms.

## Author Contributions

LD: study design and manuscript writing. HF: study design, data collection, and manuscript writing. JL: study design, data collection, and manuscript writing. CH: literature review.

## Conflict of Interest Statement

The authors declare that the research was conducted in the absence of any commercial or financial relationships that could be construed as a potential conflict of interest.
